# Assessment of the Efficacy of Olive Leaf (*Olea europaea* L.) Extracts in the Treatment of Colorectal Cancer and Prostate Cancer Using In Vitro Cell Models

**DOI:** 10.3390/molecules26134069

**Published:** 2021-07-03

**Authors:** Sarah Albogami, Aziza M. Hassan

**Affiliations:** Department of Biotechnology, College of Science, Taif University, P.O. Box 11099, Taif 21944, Saudi Arabia; a.hasn@tu.edu.sa

**Keywords:** olive leaf extract, colorectal cancer, prostate cancer, chlorogenic acid

## Abstract

Cancer is one of the most serious public health issues worldwide, ranking second only to cardiovascular diseases as a cause of death. Numerous plant extracts have extraordinary health benefits and have been used for centuries to treat a variety of ailments with few side effects. Olive leaves have a long history of medicinal and therapeutic use. In this study, the anti-cancer properties of an olive leaf extract were investigated in vitro using colorectal and prostate cancer cell lines (HT29 and PC3, respectively). A high-performance liquid chromatography analysis showed that the olive leaf extract contained a high chlorogenic acid content. Accordingly, chlorogenic acid may be related to the observed effects of the aqueous extract on cancer cells, including increased inhibition of cancer cell growth, migration, DNA fragmentation, cell cycle arrest at the S phase, reactive oxygen species (ROS) production, and altered gene expression. The effects of the extracts were greater in HT29 than in PC3 cells. These results suggest that chlorogenic acid, the main constituent in the olive extract, is a promising new anti-cancer agent. Further analyses should focus on its in vivo effects on colorectal tumor models, both alone and in combination with established agents.

## 1. Introduction

Cancer is widely regarded as one of the major human health issues on a global scale, ranking second only to cardiovascular diseases as a cause of death [1]. According to the World Health Organization, there will be approximately 30 million cases by 2050 [2]. Cytotoxic chemotherapy drugs are the gold standard for cancer treatment [3]. These drugs specifically damage cells that proliferate rapidly, including cancer and normal cells [4]. However, the efficacy and adherence to conventional chemotherapy are frequently restricted by adverse effects, such as hair loss [5], gastrointestinal complaints [6], nervous system toxicity [7], nephrotoxicity [8], and chemoresistance [9]. These conventional chemotherapeutic agents are still used to treat various cancers, such as colorectal, prostate, lung, and breast [10], emphasizing the importance of continued drug research.

Numerous plant extracts have incredible health benefits and have been used to treat a variety of ailments with few side effects since ancient times [11]. There is some encouraging clinical research on the effects of certain medicinal plant-based phenolic compounds on cancer, with a particular emphasis on metabolism, oral bioavailability, and pharmacokinetics [12,13,14]. Numerous phytochemical compounds with potent anti-cancer, anti-oxidant, and anti-inflammatory properties have been reported. In particular, polyphenols have well-established effects on carcinogenicity, and their medical benefits have generated considerable interest [15,16]. In vivo and in vitro studies have shown that polyphenols may protect against chronic diseases and revealed an inverse relationship between polyphenol intake and the risk of cancer formation [17,18,19,20]. 

The evergreen olive tree has a high polyphenol content and anti-oxidative properties [21]. Its fruits and leaves are widely consumed worldwide [22,23,24]. The olive tree (*Olea europaea* L.) has been cultivated in numerous parts of the world since ancient times [25,26,27]. Olive leaves (OLs) have a long history of therapeutic and medicinal use [28]. OLs are a rich source of valuable polyphenols, whereas olive fruits are good sources of oleuropeosides, flavonols, and flavones [29,30,31,32]. Numerous studies have established that the bioactive components in OLs possess beneficial properties, including antioxidant, antimicrobial, and antitumor characteristics [28,33,34,35,36,37]. Accordingly, OLs are a natural, inexpensive, and readily available source of phenolic compounds [38]. Extraction is the first step in analyzing and utilizing active components of medicinal plants [39]; thus, the development of appropriate OL extractive techniques is critical for increasing the yield of these bioactive components [28,40,41]. Numerous extraction methods have been used to isolate high-quality bioactive molecules, including digestion, percolation, infusion, Soxhlet extraction, maceration, and microwave-assisted extraction, although each has its limitations [42].

The precise effects of OL extracts and the components contributing to these effects are not clearly established. In this study, the anti-cancer effects of OL extracts were evaluated in vitro using colorectal and prostate cancer cell lines (HT29 and PC3, respectively).

## 2. Results

### 2.1. HPLC Analysis of Aqueous OL Extract 

**Figure 1:**
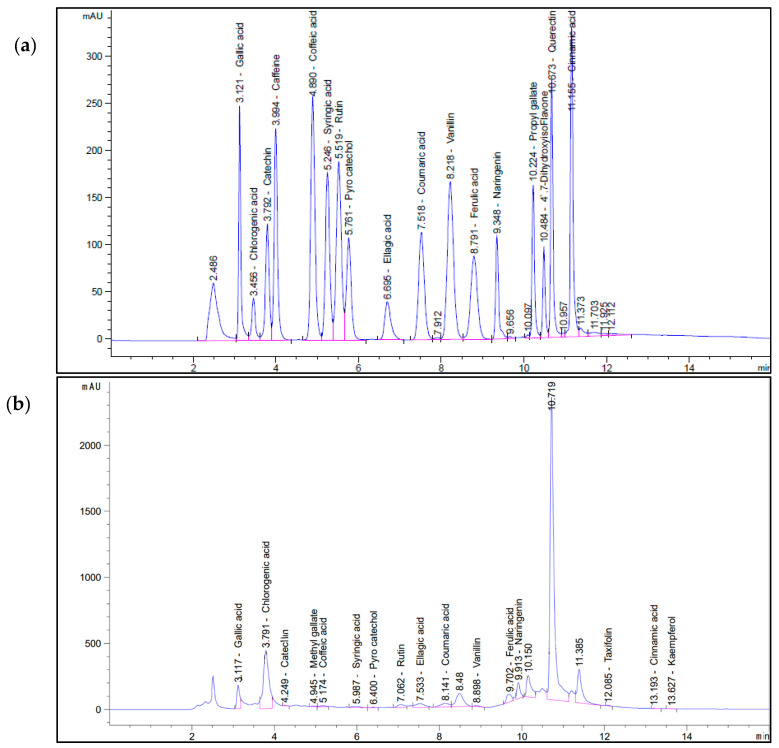
HPLC chromatogram of (**a**) standard polyphenols compounds and (**b**) the different polyphenolic components of aqueous olive leaf (AOL) extract. Conditions: At a flow rate of 1 mL/min, the mobile phase consisted of (A) water and (B) 0.02% trifluoroacetic acid in acetonitrile. Phase (A) was adjusted stepwise as follows: 0 min (80%), 0–5 min (80%), 5–8 min (40%), 8–12 min (50%), and 12–14 min (80%), followed by monitoring at 280 nm. Approximately 10 µL of each sample solution was injected, and the column temperature was maintained at 35 °C. Retention times are indicated above each peak.

**Figure 2:**
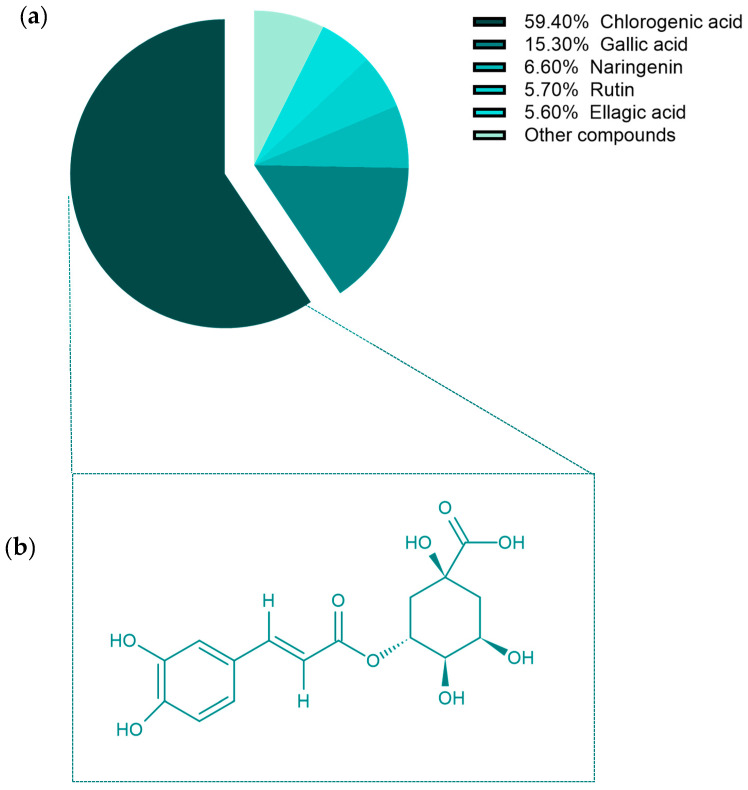
(**a**) Percentage of total polyphenols, and (**b**) Chemical structures of chlorogenic acid.

The HPLC analysis utilized here separated most of the phenolic compounds considerably well (Figure 1), with 16 clear and sharp distinct peaks obtained using the standards; these peaks for the standards were used to determine the concentration of each phenolic compound in the extract. Gallic acid, naringenin, rutin, and ellagic acid were detected at concentrations of 4.15 ± 0.83, 1.82 ± 0.56, 1.56 ± 0.61, and 1.55 ± 0.43 mg/g in aqueous OL (AOL) extract using the HPLC analysis of phenols and flavonoids (Table 1 and Figure 1). Chlorogenic acid was present at the highest concentration (i.e., 16.11 ± 1.35 mg/g), representing 59.4% of the total content of polyphenolic constituents. The overall polyphenolic content of the OL extract was 27.138 mg/g and chlorogenic acid accounted for a substantial proportion of it (Figure 2).

### 2.2. Cytotoxicity of AOLs on Cell Lines

**Figure 3:**
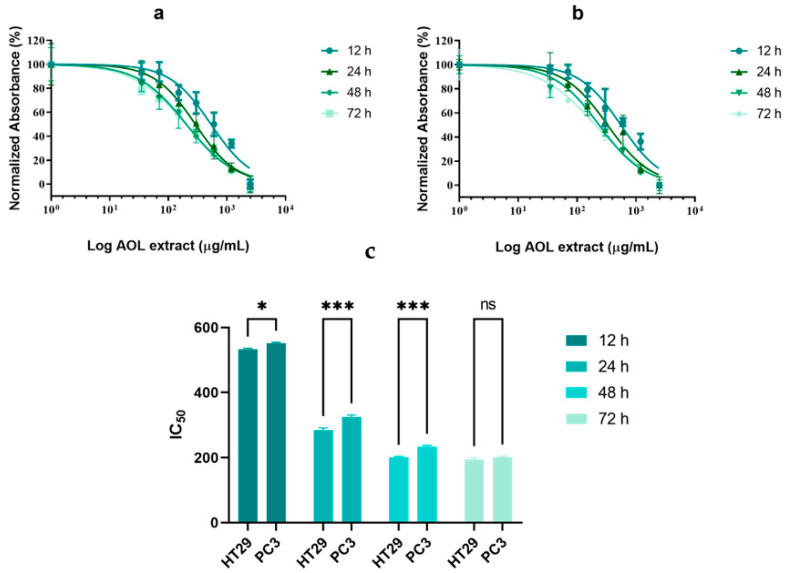
Cellular cytotoxicity of aqueous olive leaf (AOL) extracts on HT29 and PC3 cells. Dose–response curves for HT29 (**a**) and PC3 cells (**b**). (**c**) Multiple comparison of IC_50_ values between cells. The plotted values represent the means ± SDs of three independent experiments. ns: *p* > 0.05, ∗ *p* < 0.05, ∗∗ *p* < 0.01, ∗∗∗ *p* < 0.001, ∗∗∗∗ *p* < 0.0001.

Figure 3, Table S1 shows a dose response curve for AOL extracts at various concentrations on HT29 (a) and PC3 cells (b) at different time points. AOL extracts showed cytotoxic effects on both the cell lines. The IC_50_ values for HT29 cells after treatment were 535.3, 289.6, 203.1, and 198.6 at 12, 24, 48, and 72 h, respectively (Figure 3c). The calculated IC_50_ values for PC3 cells after treatment were 553.8, 328.8, 236.6, and 203.9 µg/mL at 12, 24, 48, and 72 h, respectively (Table 1). The IC_50_ values of the extract were significantly lower for HT29 cells than for PC3 cells at 12 h (*p* < 0.05), 24 h (*p* < 0.001), and 48 h (*p* < 0.001), with no significant differences observed at 72 h between the cell lines (Figure 3c). Therefore, the IC_50_ value for each cell line after 48 h was used for further analyses.

### 2.3. AOL Extract Significantly Suppressed the Cell Cycle Progression in the HT29 Cell Line

**Figure 4:**
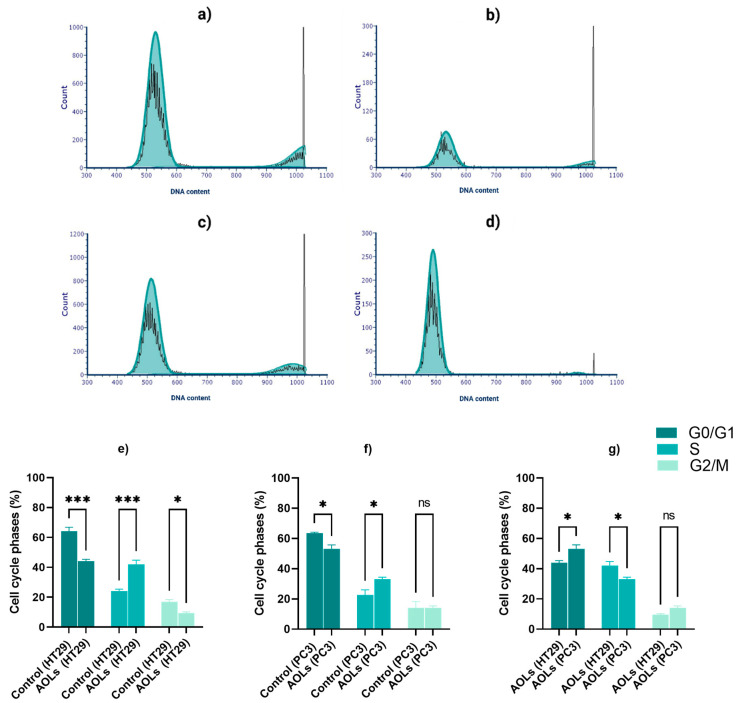
Effect of aqueous olive leaf (AOL) extract on the cell cycle phases in HT29 and PC3 cell lines. Cell cycle phase histogram for (**a**) untreated HT29 cells, (**b**) HT29 cells treated with AOL extract, (**c**) untreated PC3 cells, and (**d**) PC3 cells treated with AOL extract. (**e**–**g**) Multiple comparisons between the cell cycle phase (%) values are presented as the means ± SDs of three independent replicates, which were analyzed by two-way ANOVA: ns: *p* > 0.05, ∗ *p* < 0.05, ∗∗∗ *p* < 0.001.

Cell cycle analysis was performed to determine the extent to which the AOL extract suppressed the viability of HT29 and PC3 cells (Figure 4a–d). The results indicated that AOL extracts arrested cells in the S phase in both the cell lines (Figure 4e,f), as after AOL extract treatment, there was a significant increase in S-phase arrest in both the lines. Furthermore, when comparing the treated cells with their respective control, we found that the increase in S-phase arrest in the HT29 cell line (*p* < 0.001) was significantly higher than that in the PC3 cell line (*p* < 0.05). When comparing the cell cycle phases in treated cells with each other (Figure 4g), we found that the increased S-phase arrest in the HT29 cell line was significantly higher than that in PC3 (*p* < 0.05).

### 2.4. Cell Migration Assay

**Figure 5:**
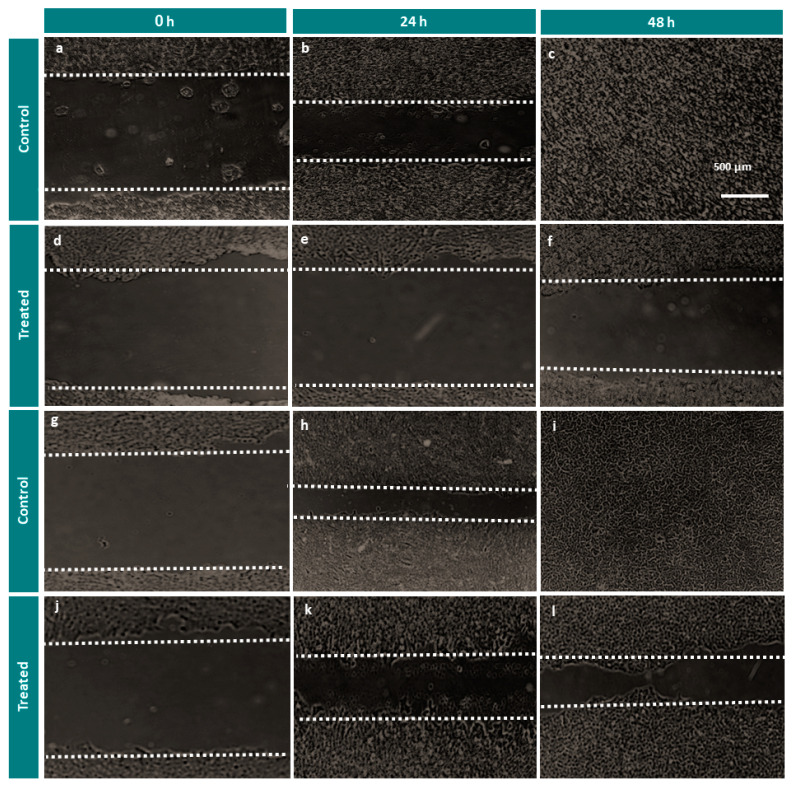
Wound-healing assays demonstrating that treatment with aqueous olive leaf (AOL) extract inhibited cell motility in HT29 and PC3 cell lines. (**a**–**c**) untreated control HT29 cells, (**d**–**f**) treated HT29 cells, (**g**–**i**) control PC3 cells, (**j**–**l**) treated PC3 cells.

**Figure 6:**
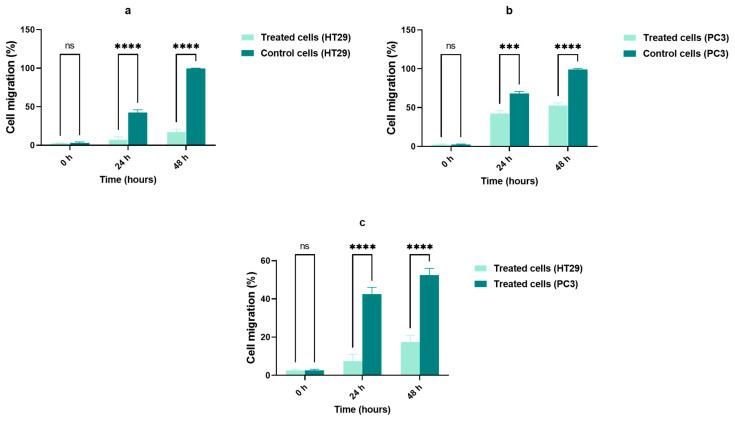
Summary of results for the wound-healing assay. Values are presented as the means ± SDs of three independent replicates and were analyzed by two-way ANOVA and multiple comparison tests. (**a**) HT29 cells treated with aqueous olive leaf (AOL) extracts vs. control (untreated cells), (**b**) PC3 cells treated with AOL extracts vs. control (untreated cells), (**c**) HT29 cells treated with AOL extracts vs. PC3 cells treated with AOL extracts. ns: *p* > 0.05, ∗∗∗ *p* < 0.001, ∗∗∗∗ *p* < 0.0001.

We evaluated cell migration by a wounding healing assay. HT29 and PC3 cell lines were treated with AOL extract at a concentration of 200 g/mL. At 24, 48, and 72 h after treatment, both the cell lines demonstrated a similar reduction in migration, as evidenced by a lower wound-healing rate than that for control cells (Figure 5a–l and Figure 6a,b). At 0 h, no significant cell migration was detected in any for the treated cell lines, as shown in Figure 4; however, control cells for both the cell lines showed complete wound healing after 72 h. As shown in Figure 6c, at 24, and 48 h post-treatment, cell migration was significantly lower for HT29 than for PC3 cells (*p* < 0.001).

### 2.5. AOL Extract Alters Cell Morphology

**Figure 7:**
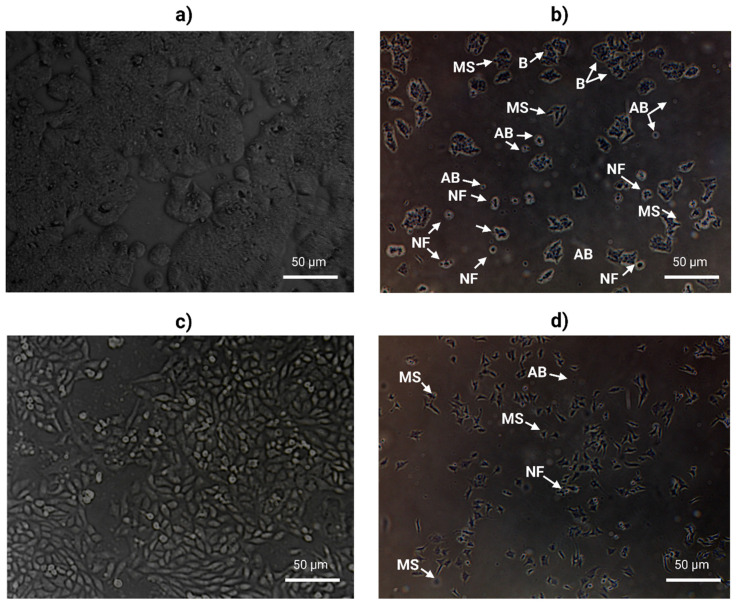
Morphological changes in HT29 and PC3 cells treated with aqueous olive leaf (AOL) extract. Cells were treated with AOL extract at their optimum IC_50_ for 48 h. (**a**,**b**) HT29, (**c**,**d**) PC3 cells. Apoptotic hallmark represented as: (B) Blebs, (MS) microtubule spikes, (NF) nuclear fragmentation, and (AB) apoptotic bodies.

To examine the changes in cell morphology following treatment with AOL extract, each cell line was treated for 48 h at IC_50_. As illustrated in Figure 7, AOL extract induced more morphological changes and a decrease in the cell number in HT29 (Figure 7b) as compared with that in PC3 (Figure 7d) relative to their respective control (Figure 7a,c). Additionally, we observed that both the cell lines treated with AOL extract exhibited several apoptotic features, including nuclear fragmentation (NF), microtubule spikes (MS), apoptotic bodies (AB), and Blebs (B).

### 2.6. DNA Fragmentation Induced by Olive Leaf Extract

**Figure 8:**
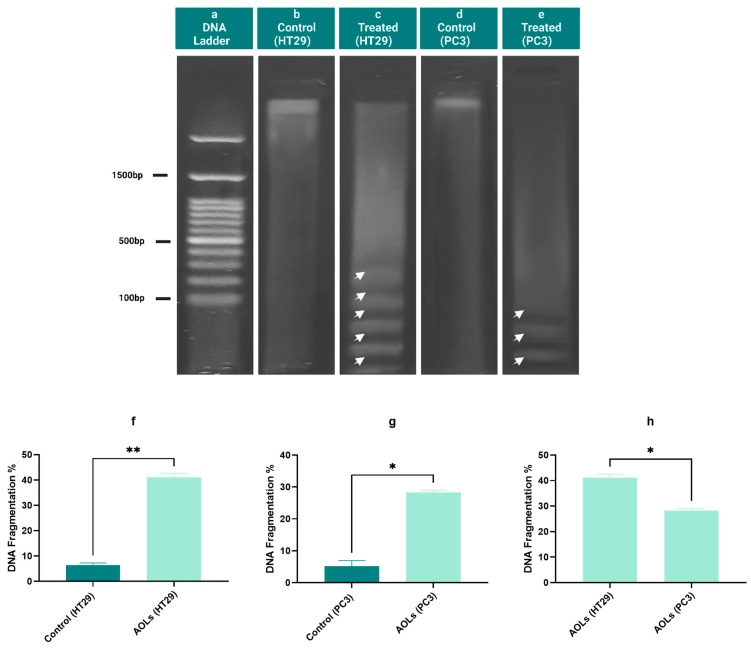
Effects of aqueous olive leaf (AOL) extract treatment on DNA fragmentation in HT29 and PC3 cells after 48 h. (**a**–**e**) DNA profiles determined by agarose gel electrophoresis. (**a**) DNA ladder, (**b**) control (untreated HT29), (**c**) treated HT29 with AOL extracts, (**d**) control (untreated PC3), (**e**) treated PC3 with AOL extracts. (**f**–**h**) DNA fragmentation percentages after 48 h (means ± SDs of three independent experiments) were analyzed by paired two-tailed *t*-tests. (**f**) HT29 cells treated with AOL extracts vs. control (untreated cells), (**g**) PC3 cells treated with AOL extracts vs. control (untreated cells), (**h**) HT29 cells treated with AOL extracts vs. PC3 cells treated with AOL extracts. ∗ *p* < 0.05, ∗∗ *p* < 0.01.

Nuclear DNA is degraded via nuclear endonucleases, and this is considered a hallmark of apoptotic cell death. A DNA fragmentation assay was used to evaluate the effects of AOL extract on cell number and cell morphology in both the cell lines. DNA fragments were detected by comparing DNA profiles by agarose gel electrophoresis, showing a ladder pattern (Figure 8). DNA fragmentation was calculated as a percentage, and the results are presented in Figure 5f–h. Treated HT29 and PC3 cells showed significantly higher rates of DNA fragmentation (*p* < 0.01, *p* < 0.05, respectively) than those in untreated cells (Figure 8f–g). However, DNA fragmentation was more prominent in HT29 cells treated with AOL extract than in PC3 cells treated with the same extract (*p* < 0.05) (Figure 5h).

### 2.7. Determination of the Expression of Genes Related to Apoptosis

**Figure 9:**
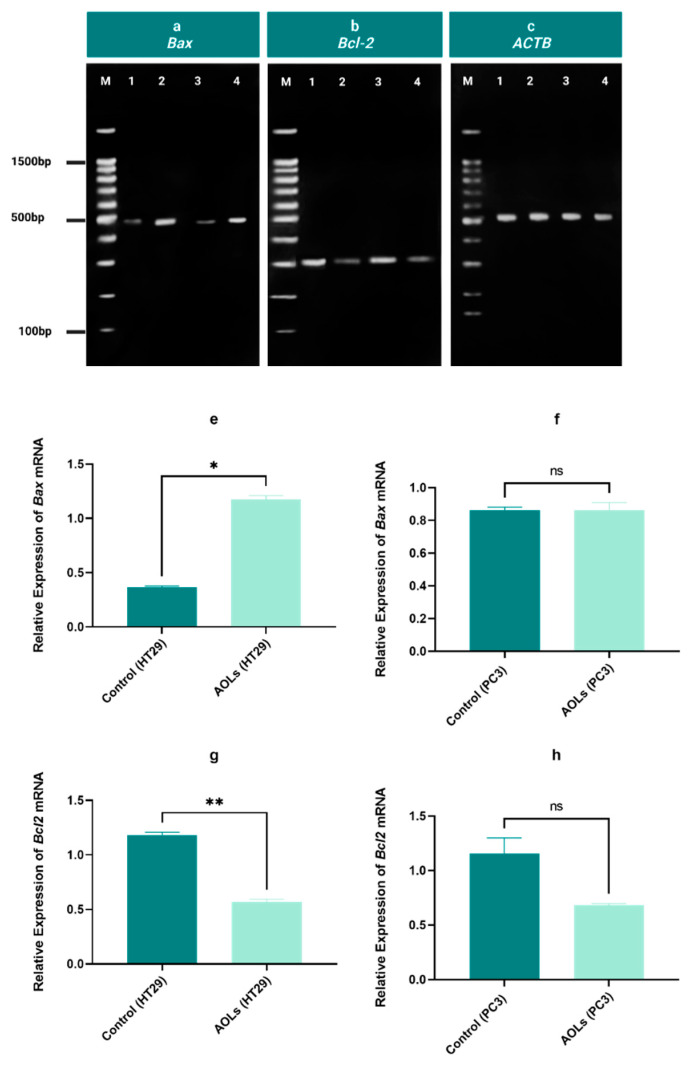
Effects of aqueous olive leaf (AOL) extract treatment on the expression of *Bax* and *Bcl2* in HT29 and PC3 cells after 48 h. (**a**–**c**) *Bax*, *Bcl2*, and *ACTB* expression profiles in both the cell lines determined by agarose gel electrophoresis. (**a**) *Bax* expression, (**b**) *Bcl2* expression, (**c**) *ACTB* expression. (M) DNA ladder, (1) control (untreated HT29), (2) treated HT29 with AOL extracts, (3) control (untreated PC3), (4) PC3 treated with AOL extracts. (**e**–**h**) Relative expression of *Bax* and *Bcl2* after 48 h (mean values ± SD of three independent experiments) was analyzed by paired two-tailed *t*-tests. (**e**) *Bax* expression in HT29 cells treated with AOL extracts vs. control (untreated cells), (**f**) *Bax* expression in PC3 cells treated with AOL extracts vs. control (untreated cells), (**g**) *Bcl2* expression in HT29 cells treated with AOL extracts vs. control (untreated cells), (**h**) *Bcl2* expression in PC3 cells treated with AOL extracts vs. control (untreated cells), ns: *p* > 0.05, ∗ *p* < 0.05, ∗∗ *p* < 0.01.

Gene expression was analyzed by quantifying the signal intensities of each band. Bands produced by amplifying cDNA of either colon or prostatic cancer cell lines and the housekeeping gene, β-actin, as a control were analyzed. The ratio of the maximum optical density of the targeted amplification product (max.OD) and the average maximum optical density of β-actin was calculated. The expression levels of *Bax* and *Bcl2* mRNA in both untreated and treated cancer cells are summarized in Figure 9. The expression level of *Bax* in HT29 cells treated with AOL extract was significantly higher than that in control cells (untreated HT29 cells) (*p* < 0.05). The expression level of *Bax* did not differ between PC3 cells treated with AOL extracts and control cells (untreated PC3 cells). The expression level of *Bcl2* in AOL-treated HT29 cells was significantly lower than that in control cells (untreated HT29 cells) (*p* < 0.01). Bcl2 expression in AOL extract-treated PC3 cells was not significantly different from that in control cells (untreated PC3 cells). These results suggest that AOL extracts have a greater effect on HT29 cells than on PC3 cells.

### 2.8. Effects of AOL Extract on the Oxidative Stress and Antioxidant Parameters

**Figure 10:**
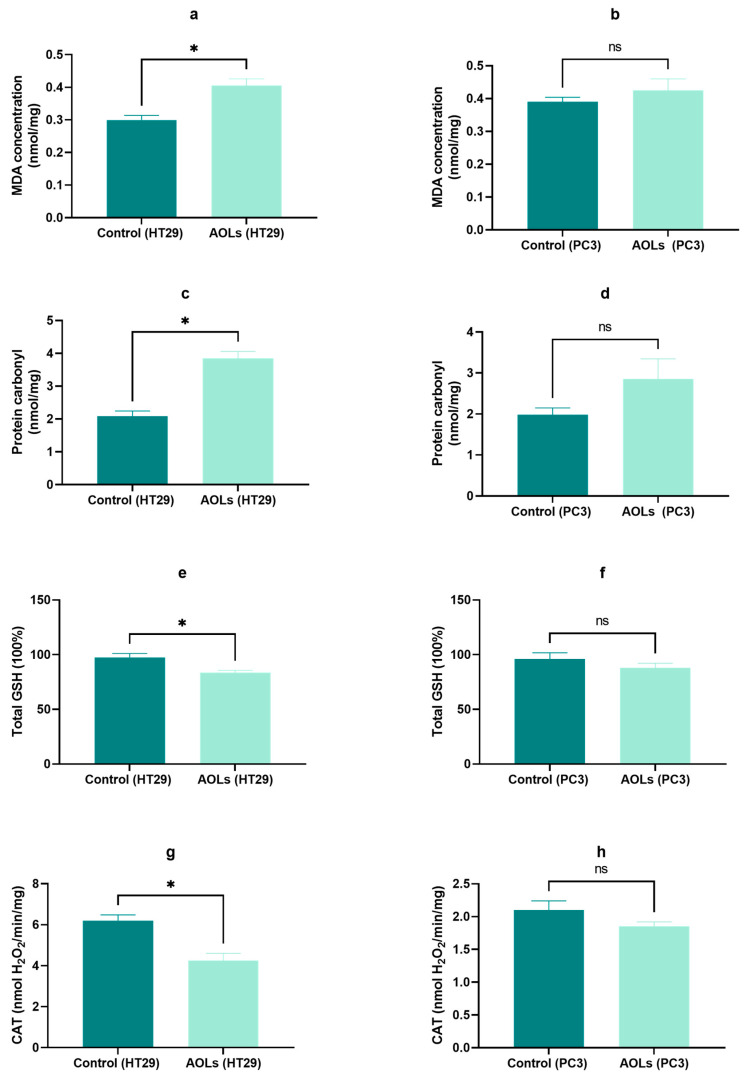
Effect of aqueous olive leaf (AOL) extract on oxidative stress and antioxidant parameters**.** (**a**,**b**) malondialdehyde (MDA) content, (**c**,**d**) protein carbonyl content, (**e**,**f**) glutathione content, (**g**,**h**) catalase activity. Values (expressed as ± SD of three independent experiments) were analyzed by paired two-tailed *t*-tests. ns: *p* > 0.05, ∗ *p* < 0.05.

The effect of AOL extract on malondialdehyde (MDA) content in in HT29 and PC3 cells was explored, and the detected MDA levels were significantly increased (*p* < 0.05) in HT29 cells treated with AOL extract for 48 h compared to that in the control cells (Figure 10a), whereas MDA levels were increased in treated PC3 cells, but not significantly (Figure 10b). This finding indicates that AOL extract promoted lipid peroxidation in HT29 cells.

Additionally, we observed an increase in the level of protein carbonyls in HT29 cells (*p* < 0.05), whereas this increase was not significant in PC3 cells treated with AOL extract as compared to control cells (Figure 10c,d). This result indicates that AOL extract accelerated the oxidation of proteins in HT29.

A significant decrease (*p* < 0.05) in the GSH and catalase levels was observed in HT29 cells treated with AOL extract compared with that in the control cells, whereas the decrease in the GSH and catalase levels observed in PC3 was not significant (Figure 10e–h). This result indicates that AOL extract reduced the antioxidant capacity of HT29 cells.

## 3. Discussion

Epidemiological studies have shown that the Mediterranean diet reduces the incidence of some types of cancer, including colon cancer [43]. Free radicals are believed to be essential for the development of cancer [44,45,46,47]. There is at least some scientific evidence suggesting that phenolic compounds have beneficial effects on human health, owing mainly to their antioxidant capacity [48,49,50]. Antioxidant components can aid in the management of diseases that are associated with oxidative stress, such as cancers [51]. Phytochemicals have anticancer properties in part owing to their ability to regulate and reduce reactive oxygen species and thus protect against oxidative stress [52]. The use of antioxidant components in cancer drug development is a source of contention owing to a dearth of conclusive results [53].

The antioxidant properties of olive phenolic compounds may affect outcomes and decrease the risk of cancer. The anti-cancer activity of *Olea europaea* L. has been demonstrated both in vitro and in vivo. The extract concentration and exposure time are likely to be critical determinants of the biological effects of compounds on cancer cell lines and other in vivo systems [54]. We observed changes in viability, proliferation, migration, DNA fragmentation, and gene expression in two cancer cell lines, HT29 and PC3, following treatment with AOLs. Ethanol extracts from Lebanese OLs induced apoptosis in leukemic cells (Jurkat cells) with an IC_50_ of approximately 4 mg/mL at 48 h and 3 mg/mL at 96 h; the OL extracts in our study exerted an anti-proliferative effect with less cytotoxicity [55]. In another study, ethanol extracts from OLs of Chemlali olive trees (the most abundant olive tree in Tunisia) at concentrations of 100 and 150 µg/mL showed antiproliferative effects against the human chronic myeloid leukemia cell line K562 (stem-like cells with high drug resistance) and these effects were mediated by the suppression of cell cycle progression, induction of apoptosis, and promotion of differentiation toward the monocyte progenitor cell lineage [56]. Another study has shown that oleuropein and its peracetylated derivative inhibit cell proliferation in two thyroid cancer cell lines, TPC-1 and BCPAP, by triggering MAP kinase and PI3K-AKT signaling pathways and exerting antioxidant effects [57]. MCF-7 and T-47D human breast cancer cell lines were used to investigate the biological effects of oleuropein and its synthetic peracetylated derivatives after cells are exposed to hydrogen peroxide, demonstrating that the peracetylated derivatives inhibit cell proliferation, as evidenced by cell cycle arrest [58]. Aqueous and methanol OL dried extracts prevent the growth of human breast adenocarcinoma (MCF-7), human urinary bladder carcinoma (T-24), and bovine brain capillary endothelial cells [59]. The antitumor activity of a hydroxytyrosol-rich OL extract has been reported in MCF-7 breast cancer cells, showing a dose-dependent inhibitory effect by arresting cells in the G-phase [60]. Furthermore, at the molecular level, treatment with OL extract increased the expression of c-jun, a member of the AP1 transcription factor family [60]. In a randomized clinical trial with patients receiving chemotherapy for cancer, it was discovered that 333 mg/mL *Olea europaea* leaf extract significantly reduced the expression of two pro-inflammatory cytokines, interleukin-1 beta (IL-1b) and TNF alpha (TNF-a), after 2 weeks and prevented the development of severe oral mucositis [61]. Another study investigated mice models of breast cancer treated for three weeks with 75, 150, or 225 mg/kg/day of olive leaf extract. The authors found that the doses of 150 and 225 mg/kg/day resulted in a significant decrease in the volume and weight of the tumor. It also increased the CAT activity and increased the activity of superoxide dismutase [62].

Our HPLC analysis showed that the extract contained a high amount of chlorogenic acid. This high chlorogenic acid content in the aqueous extract may explain the inhibition of cancer cell growth, migration, DNA fragmentation, and gene expression. Chlorogenic acid causes a phenotypic transformation, specifically in the process of cell differentiation [63]. Huang et al. indicated that the effects of AOLs differ among cancer cell lines. Accordingly, the effect of AOLs depends on the cell type [63]. Chlorogenic acid alone could have effects similar to the results obtained here, as the effect of chlorogenic acid was investigated in the human colon cancer cell lines HCT116 and HT29. They found that chlorogenic acid in both the cell lines could inhibit cellular viability, induced the production of reactive oxygen species (ROS) and cell cycle arrest at the S phase, and inhibited the activation of extracellular signal-related kinase [64]. In vitro and in vivo studies showed that chlorogenic acid is degraded to caffeic acid, which is then transformed into various catabolic derivatives, including dihydrocaffeic acid [65,66,67]. In another study, the anticancer potential of dihydrocaffeic acid as a metabolite of chlorogenic acid using several cancer cell lines, including MCF7, HepG2, PC3, and HCT116, was explored. The authors found dihydrocaffeic acid to be significantly more cytotoxic to all cancer cell lines except Hep-G2, with a cytotoxic dose ranging between 162 and 390 M in PC3 [68]. In a study on the effectiveness of chlorogenic acid as a chemosensitizer in inhibiting tumor growth via metabolic pathways, this agent had the ability to stimulate and suppress many vital signaling pathways in cancer metabolism [69]. Chlorogenic acid might exert antitumor effects by modulating mTOR complex 2 signaling and interrupting the organization of filamentous actin [70]. Chlorogenic acid improves the efficacy of 5-fluorouracil in human hepatocellular cancer cell lines (HepG2 and Hep3B) by inhibiting extracellular signal-regulated kinase ERK via excessive reactive oxygen species synthesis [71].

The typical level of cholorogenic acid in human plasma has been determined using liquid chromatography–mass spectrometry (LC–MS) and ranged from 10 to 2000 ng/mL [3]. Ultra-performance liquid chromatography (UPLC) indicated a range from 5 to 2000 ng/mL for human plasma and 50 to 20,000 ng/mL for human urine [4].

**Figure 11:**
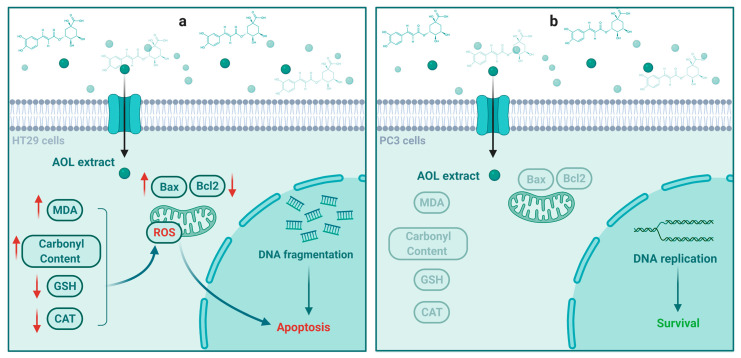
Molecular mechanism suggested for aqueous olive leaf (AOL) extract in triggering apoptosis in (**a**) HT29 and (**b**) PC3 cancer cells.

Our current findings indicate AOL extract induces significant apoptosis in HT29 cells by decreasing Bcl2 and increasing the Bax-mediated mitochondrial pathway, which involves the arrest of the cell cycle in the S phase, the production of ROS, DNA fragmentation. As a consequence, caspases are activated and apoptosis is induced, as shown Figure 11. However, we found that AOL extract did not affect PC3 as significantly as HT39 cells.

Furthermore, it is well established that the phosphatidylinositol 3-kinase/protein kinase B (PI3K/AKT) pathway is a critical survival pathway that is triggered in cancer [72,73]. Previous studies have shown a correlation between the activation of the PI3K/AKT signaling pathway and an increase in invasion and migration of human cancer cells [73]. A study by Yan and his colleagues showed that oleuropein (a type of polyphenol that occurs naturally in olive oil), stimulates the synthesis of ROS in hepatocellular carcinoma cells, and when ROS was removed, the level of phosphorylated AKT was increased leading to reduced activation of apoptosis [74]. This led us to hypothesize that PI3K/AKT may be considered as a critical mediator of HT29 cell apoptosis induced by AOL extract. Therefore, further investigation is required to find the exact molecular mechanism that led to the AOL extract-mediated induction of apoptosis in HT29 cells, and it is important to compare the results with commercially available chlorogenic acid. 

The migration and proliferation of human tumor cells are inhibited in vitro after exposure to oleuropein, one of the components of olive extract [75,76]. Hamadi and Castellon showed that oleuropein results in the rounding of cancer cells irreversibly, inhibiting motility, replication, and invasiveness, but similar effects were reversible in normal cells when the therapy was removed [76]. In their study, they showed that oleuropein is expected to enter the cell via glucose transporters because of its glucose moiety (GLUTs). The anti-proliferative effect of oleuropein is reduced when the glucose moiety is removed with glucosidase, indicating that the glucose moiety is at least largely responsible for the entry of oleuropein into the cell [77]. Elevated glucose absorption and increased expression of many GLUT isoforms have been linked to human cancers. Because normal cells have minimal GLUT expression, this explains how normal cells can counteract the rounding effects of oleuropein therapy [78].

The mechanism by which olive leaf-induced ROS production affects cell viability is both intriguing and contentious, as olive leaves are capable of stimulating ROS accumulation in HepG2 cells [54]. It was shown that oleuropein, one of the constituents of leaves of the olive, could be antioxidant and pro-oxidant depending on the kind of cell used (cancer or normal), the time of treatment, and the concentration used. It has been demonstrated that oleuropein appears to have the greatest antioxidant activity in normal hepatic tissue [79,80], whereas it induces a ROS-mediated apoptotic pathway in human leukemia and breast cancer cell lines [81,82].

The findings of this study provide strong evidence for the status of AOL extract as a powerful antioxidant. Our results further indicate that AOL extract exhibits different impacts in vitro, based on the type of cell used; the differences in the activity of the AOL extract raises concerns about the precarious balance between oxidative and antioxidant states and demonstrates the risks of drawing conclusions based solely on in vitro results to characterize the true properties of the AOL extract.

Taken together, these findings suggest that mitochondrial and oxidative pathways are involved in the apoptosis induced by AOL extract in HT39 cells. Furthermore, the apoptotic effect of AOL extract is most likely unique and may vary according to the mechanism of each component, the manner in which they were extracted, and the cell lines used. Additional mechanisms, however, are almost certainly involved and further investigation are required to fully understand these mechanisms.

## 4. Materials and Methods

### 4.1. Plant Material Collection and Processing

Fresh OLs (*O. europaea* L.) were collected from the Al Shafa Mountains, Taif, Saudi Arabia in March 2020. Botanists from the Department of Biotechnology at Taif University verified the authenticity of plant leaves. The leaves were cleaned, dried, ground, and stored at 20 °C until use.

### 4.2. Extract Preparation

Leaves were washed to eliminate particles, such as dust, before being dried in an oven at 45 °C for 48 h. The finely ground sample (5 g) was extracted for 45 min with 250 mL of boiling distilled water and filtered through Whatman paper. Finally, the aqueous extract was frozen and lyophilized using a freeze-dryer (ALPHA, 1-2 LD plus, Osterode am Harz, Germany). The anticancer activity of the lyophilized extract was tested after it was dissolved in water at a concentration of 50 mg/mL.

### 4.3. HPLC Analysis of Aqueous Olive Leaf Extract

An Agilent 1260 HPLC series was used to analyze the AOLs. A 100-pore size C18 column (L I.D. 4.6 mm × 250 mm, 5 m particle size) was used for separation. At a flow rate of 1 mL/min, the mobile phase consisted of (A) water and (B) 0.02% trifluoroacetic acid in acetonitrile. Phase (A) was adjusted stepwise as follows: 0 min (80%), 0–5 min (80%), 5–8 min (40%), 8–12 min (50%), and 12–14 min (80%), followed by monitoring at 280 nm. Approximately 10 µL of each sample solution was injected, and the column temperature was maintained at 35 °C. The mean values and standard errors (SEs) are reported.

The AOL HPLC chromatogram was compared to a standard mixture of identified compounds. By comparing the retention times of the compounds in the AOL extract to the corresponding standards, the compounds were identified.

### 4.4. Cell Culture

Two human cell lines were used: the colon cancer cell line HT29 and the prostate cancer cell line PC3 (American Type Culture Collection, Manassas, VA, USA). All cells were routinely cultured in Dulbecco’s modified Eagle’s medium (Sigma Aldrich, St. Louis, MO, USA) supplemented with 10% fetal bovine serum (Gibco Invitrogen, Gaithersburg, MD, USA), 4 mM l-glutamine (Sigma Aldrich), 1% penicillin/streptomycin (Sigma Aldrich), and 10 mM HEPES (Gibco). The cells were grown at 37 °C in a humidified incubator containing 5% CO_2_.

### 4.5. Cytotoxicity Assay (MTT Assay)

The cells were grown in 96-well plates at a density of 1 × 10^5^ cells per well and incubated for 24 h to allow them to adhere and proliferate. Media were aspirated and serum-free media (Gibco) with various concentrations of AOLs (35, 70, 150, 300, 600, 1200, and 2500 µg/mL) were added to each well, followed by incubation for 12, 24, 48, and 72 h. At each time point, media were discarded, cells were washed with PBS, and 50 µL of MTT solution (10 mg/mL; Sigma Aldrich) and 50 µL of serum-free media were added to each well. The plates were incubated for 3 h at 37 °C, and 150 µL of DMSO (MTT solvent) was added to each well. The plates were wrapped with foil and shaken for 15 min on an orbital shaker at 25 °C. Absorbance was measured at OD = 590 nm within 1 h using a microplate reader (Spectramax Plus 384; Molecular Devices, San Jose, CA, USA).

### 4.6. Cell Cycle Distribution Analysis 

Untreated cells and cells that were treated with AOL extract at their optimum IC_50_ dose for 48 h were trypsinized and centrifuged for 5 min at 300× *g*. The supernatant was removed, and the pellet resuspended in medium, then cells were centrifuged again, and the supernatant was removed. 1 × 10^6^ cells were resuspended in PBS and fixed in 70% ethanol on ice for 2 h. The ethanol-suspended cells were centrifuged at 300× *g* for 5 min, the supernatant was removed, and the cell pellets were suspended in 5 mL PBS and centrifuged at 300× *g* for 5 min. Then, the supernatant was removed, and the cell pellet suspended in 1 mL propidium iodide staining solution (Cayman Chemical, Ann Arbor, MI, USA) and incubated for 20 min at 37 °C in the dark. Cell florescence was measured using a flow cytometer (BD Biosciences, San Jose, CA, USA), and histograms were analyzed using the FCS express 7 cytometry software (De Novo Software, Pasadena, CA, USA).

### 4.7. Migration Assay

Cells (4 × 10^4^ cells/well) were seeded in a well plate to reach confluence. A wound was created in the middle of each well using a sterile 200-µL filter pipette tip. Media were aspirated from each well and replaced with fresh media. Each cell line was treated with AOLs at 200 µg/mL and cell migration was assessed after 0, 24, and 48 h. Images of each cell line at each time point after treatment were obtained using the EVOSTM FL Cell Imaging System (Life Technologies, Carlsbad, CA, USA) at a magnification of 40× and ImageJ (National Institutes of Health, Bethesda, MD, USA) was used to convert each captured image into a binary image. Values are presented as the means ± standard deviations of three independent replicates at each time point.

### 4.8. Assessment of Morphological Changes

Cells were cultured at a density of 2 × 10^5^ cells, then each cell line was treated with AOL extract at their optimum IC_50_ for 48 h. The changed in morphological level was investigated in treated cells compared to untreated in both cell lines. EVOSTM FL cell imaging system was used to image the cells (Life Technologies, Carlsbad, CA, USA).

### 4.9. DNA Fragmentation Assay 

Apoptosis is measured by inter-nucleosome DNA fragmentation. DNA fragmentation from extracted genomic DNA was evaluated using a Bio-Vision DNA Ladder Detection Kit (Bio-Vision, Life Science SOURCE, Milpitas, CA, USA), following the manufacturer's instructions. After suspending the DNA pellet in a buffer, the samples were separated on a 1.5% agarose gel. The DNA bands were analyzed and evaluated under UV light. Diphenylamine was used to calorimetrically assign fragmented DNA quantities at 575 nm, as defined by Burton (1956), and updated by Perandones et al. (1993).

### 4.10. Gene Expression Analysis

RNA was isolated using TRIzol reagent and its integrity was tested by gel electrophoresis to analyze the expression profiles of genes related to apoptosis in cancer cells subjected to different treatments. DNase digestion was performed on the isolated RNA before it was used to produce cDNA using transcription reagents from iNtRON Biotechnology (Maxime RT PreMix) (Korea). PCRs were carried out using a thermal cycler (PXE 0.5 Thermo) and the primer pairs described in Table 2, according to Sun et al. (2013). The PCR amplification products were visualized on a 1.5% agarose gel using a Trans-illuminator UV. To measure signal intensities, the DNA bands were scanned using Gel-Pro (version 3.1 for Windows 3) (Informer Technologies, Los Angeles, CA, USA). To standardize the initial variance in the sample as an endogenous control gene for reaction productivity, the ratio of the levels of the target gene to ACTB was calculated (Raben et al., 1996).

### 4.11. Oxidative Stress and Antioxidant Determination

#### 4.11.1. Cells Supernatant Preparation

Each cell line was cultured at a density of 2 × 10^6^ cells for 24 h. Then, cells were treated with AOL extract at their optimum IC_50_ dose for 48 h, and untreated cells were used as control. Cells were harvested by centrifugation at 500× *g* for 10 min, and then, the medium was discarded. About 100 μL of cold lysis buffer was added to each cell pellet, which was then incubated on ice for 10 min, the cell mixture was centrifuged at 13,000× *g* for 10 min at 4 °C and the supernatant collected. 

#### 4.11.2. Determination of Malondialdehyde (MDA) 

The reaction mixture was prepared by mixing the following: 200 μL of each sample’s supernatant, 250 μL trichloroacetic acid (TCA) with a purity of ≥99% (purchased from Sigma Aldrich (St. Louis, MO, USA)), 12.5 μL of butylated hydroxytoluene with a purity of ≥99% (Sigma Aldrich), and 400 μL of PBS. The mixture for each sample was incubated at 4 °C for 120 min, and then centrifuged at 13,000× *g* for 10 min. The obtained supernatant from each sample was mixed with 125 μL of thiobarbituric acid (TBA) with a purity of ≥99% (BDH Chemical, Poole, England) and incubated at 95 °C for 60 min. The reaction was maintained for 10 min. Absorbance was measured immediately at OD 532 nm on a microplate reader (Pharmaspec, Shimadzu, Japan), as described in [83]. The obtained value was expressed as nmol/mg.

#### 4.11.3. Determination of Protein Carbonyl Content

A protein carbonyl content assay kit was used to measure protein oxidation according to the manufacturer’s protocol (abcam). The assay is based on the reaction of dinitrophenylhydrazine (DNPH) with protein carbonyls. The OD was measured at ~375 nm on a microplate reader (Pharmaspec). The obtained value was expressed as nmol/mg.

#### 4.11.4. Determination of Glutathione (GSH) Content

A glutathione assay kit (Trevigen, Gaithersburg, MD, USA) was used according to the manufacturer’s protocol. The absorbance was measured immediately at OD 402 nm on a microplate reader (Pharmaspec). The glutathione content was normalized to the protein content using a BCA kit (BioVision, Milpitas, CA, USA). The obtained value was expressed as total GSH (100%).

#### 4.11.5. Determination of Catalase (CAT) Content

The reaction mixture was prepared by mixing the following: 100 μL of each sample’s supernatant, 1 mL of hydrogen peroxide, and 1.9 mL (0.05 M, pH 7.0) of phosphate buffer. Absorbance was measured immediately at OD 240 nm on a microplate reader (Pharmaspec, Shimadzu, Japan), as described in [84]. The obtained value was expressed as nmol H_2_O_2_/min/mg.

### 4.12. Statistical Analysis

Statistical analyses were conducted using GraphPad Prism 9.1.1 (La Jolla, CA, USA). Two-way ANOVA was used to analyze cell migration assay data and cell cycle phases. A two-tailed paired *t*-test was used to analyze DNA fragmentation and gene expression, and oxidative stress and antioxidant determination results. IC_50_ values were evaluated by a dose–response curve. Differences between groups were considered statistically significant at *p* ˂ 0.05. All experiments were performed in triplicate.

## 5. Conclusions

The results of the current study suggest that AOL extract is rich in chlorogenic acid. This extract more strongly inhibited the growth of the colorectal cancer cell line HT29 than the prostate cancer cell line PC3. These findings suggest that OLs are a promising source of chlorogenic acid for the development of a new therapeutic agent for colorectal cancer. Additional research utilizing pure chlorogenic acid in vivo will shed additional light on the efficacy of this compound alone or in combination with currently available treatments. Furthermore, additional experiments are necessary to comprehensively characterize its activity and molecular mechanisms of action.

## Figures and Tables

**Table 1:** Polyphenolic compounds in leaf extracts (µg/g).

Plant Extract	Aqueous Olive Leaf Extract (AOLs)	
Polyphenol Compounds	Retention Time (min)	Quantification (µg/g)
Gallic acid	3.117	4152.31
Chlorogenic acid	3.791	16111.86
Catechin	4.249	73.79
Methyl gallate	4.945	27.95
Caffeic acid	5.174	205.77
Syringic acid	5.987	229.79
Pyro catechol	6.400	87.82
Rutin	7.062	1558.40
Ellagic acid	7.533	1544.58
Coumaric acid	8.141	362.44
Vanillin	8.898	74.47
Ferulic acid	9.702	819.82
Naringenin	9.913	1817.55
Taxifolin	12.085	87.23
Cinnamic acid	13.193	1.49
Kaempferol	13.627	11.09
Total polyphenolic content	27,166,36	

**Table 2:** Sequences of oligonucleotide primers used for PCR.

Gene Symbol	ID	Sequence (5′–3′)		Gene Length (bp)
*ACTB*	ENSG00000075624	Forward primer	GTCACCAACTGGGACGACATG	510
		Reverse primer	GCCG TCAGGCAGCTCGTAGC	
*BCL2*	ENSG00000171791	Forward primer	GTGGAGGAGCTCTTCAGGGA	304
		Reverse primer	AGGCACCCAGGGTGATGCAA	
*BAX*	ENSG00000087088	Forward primer	GGCCCACCAGCTCTGAGCAGA	477
		Reverse primer	GCCACGTGGGCGTCCCAAAGT	

*ACTB*, actin beta; *BCL2*, BCL2 apoptosis regulator; *BAX*, BCL2 associated X; apoptosis regulator, *VEGFA*; vascular endothelial growth factor A.

## Data Availability

Not applicable.

## Supplementary Materials

The following are available online, Table S1: Log (inhibitor) vs. normalized response for HT29 and PC3.

## Sample Availability

Samples of the AOL extract are available from the authors.
