# Supplementary material for: Assessment of the Efficacy of Olive Leaf (Olea europaea L.) Extracts in the Treatment of Colorectal Cancer and Prostate Cancer Using In Vitro Cell Models
Source: Molecules. 2021 Jul 3;26(13):4069. doi: 10.3390/molecules26134069 (PMC8272070; doi:10.3390/molecules26134069)
Supplement: Supplementary file 1 [file molecules-26-04069-s001.zip › molecules-1263313-supplementary.pdf]

Supplymentry materials

HT29					
**log(inhibitor) vs. normalized response**		12 hours	24 hours	48 hours	72 hours
**Best-fit values**	**$LogIC_{50}$**	2.729	2.462	2.308	2.298
	**IC_{50}**	535.3	289.6	203.1	198.6
**Std. Error**	**$LogIC_{50}$**	0.04658	0.04389	0.04751	0.04205
**95% CI (asymptotic)**	**$LogIC_{50}$**	2.629 to 2.829	2.368 to 2.556	2.206 to 2.410	2.208 to 2.388
**Goodness of Fit**	**Degrees of Freedom**	14	14	14	14
	R^2	0.9469	0.9591	0.9567	0.9668

PC3					
**log(inhibitor) vs. normalized response**		12 hours	24 hours	48 hours	72 hours
**Best-fit values**	**$LogIC_{50}$**	2.743	2.517	2.374	2.309
	**IC_{50}**	553.8	328.8	236.6	203.9
**Std. Error**	**$LogIC_{50}$**	0.04851	0.05282	0.03524	0.04839
**95% CI (asymptotic)**	**$LogIC_{50}$**	2.639 to 2.847	2.404 to 2.630	2.299 to 2.450	2.206 to 2.413
**Goodness of Fit**	**Degrees of Freedom**	14	14	14	14
	R^2	0.9428	0.9430	0.9741	0.9571

Table S1 Log (inhibitor) vs. normalized response for HT29 and PC3
